# Comparison of CNS vasculitis in children and adults; is this the same disease entity

**DOI:** 10.1186/1546-0096-10-S1-A83

**Published:** 2012-07-13

**Authors:** Marinka Twilt, Carlo Salvarani, Tania Cellucci, Gene G Hunder, Susanne M Benseler

**Affiliations:** 1 Arcispedale S. Maria Nuova, Reggio Emilia, Italy; 2 Hospital for Sick Children, Toronto, ON, Canada; 3 Mayo Clinic, Rochester, MN, USA

## Purpose

Primary vasculitis of the central nervous system is a newly recognized, devastating inflammatory disease restricted to the brain and spinal cord. Previously healthy children and adults develop life-threatening neurological deficits including stroke and seizures. Within CNS vasculitis distinct disease entities have been identified in different age groups. The aim was to describe the presenting features of children and adults with CNS vasculitis, to compare distinct entities and their treatment regimens between adult and children and to analyze disease progression and/or relapse.

## Methods

Two cohorts of consecutive patients with primary CNS vasculitis based on Calabrese criteria diagnosed over a 21 year period and followed at tertiary vasculitis centers were identified: 1) adult patients (Mayo Clinic, Rochester) and 2) pediatric patients (Sickkids, Toronto). Patient demographic characteristics, presenting clinical features, neuroimaging characteristics, brain pathology results, clinical course including progression and relapse and treatment regimens were recorded. Characteristics of cohorts were analyzed using descriptive statistics. Univariate analysis compared distinct disease phenotypes and treatment regimens. Kaplan Meier survival analysis was performed to determine survival after CNS vasculitis diagnosis.

## Results

A total of 227 patients were included in the study; 101 adult patients and 126 pediatric patients. In children the median age was 8 years (1-17 years) and in adults 47 years (17-84 years). Pediatric patients showed a male predominance (F: M = 1:1.5) in contrast with the adult cohort (F:M = 1.3: 1). Diagnosis was established on angiography only in 88 pediatric and 52 adult patients, and on brain biopsy with negative angiography in 29 pediatric and 8 adult patients. Vessels involved were: in children 35 small vessel and 91 large vessel, in adults 22 small vessel, 6 large vessel and 48 small and large vessels. Brain biopsy: performed in 49% of adult patients with a male predominance (F: M = 1:1.2) compared to 25% in children with a female predominance ( F:M = 1.9:1). Histology showed only lymphocytic inflammation in children and predominantly a granulomatous pattern in adults (18 granulomatous, 8 lymphocytic, 5 acute necrotizing). Treatment strategies: in children prednisone in 59 (47%) and cyclophosphamide in 34 (27%), in adults prednisone in 97 (96%) and cyclophosphamide in 46 patients (46%). Relapse or progression of the disease was seen in adults in 26% and in children in 15%.

## Conclusion

Distinct differences are present in the spectrum of primary CNS vasculitis in adults and children. Biopsy only or angiography only confirmed disease shows different gender predominance in adults compared to children. Adults are treated more frequently with steroids and cyclophosphamide, however relapse rates are lower in children.

## Disclosure

Marinka Twilt: None; Carlo Salvarani: None; Tania Cellucci: None; Gene G. Hunder: None; Susanne M. Benseler: None.
